# The Need for Culturally Tailored Cardiac Rehabilitation Programmes for Underserved, Culturally and Linguistically Diverse Populations in Australia

**DOI:** 10.1002/nop2.70536

**Published:** 2026-04-02

**Authors:** Lemma N. Bulto

**Affiliations:** ^1^ Health Evidence Synthesis, Recommendations, and Impact (HESRI), School of Public Health, Faculty of Health and Medical Science The University of Adelaide Adelaide South Australia Australia; ^2^ Caring Futures Institute, College of Nursing and Health Sciences Flinders University Adelaide South Australia Australia

Cardiovascular disease (CVD) remains a leading cause of mortality in Australia, disproportionately affecting underserved and priority populations such as Aboriginal and Torres Strait Islander peoples, migrants and refugees, and those from socioeconomically disadvantaged backgrounds (Australian Bureau of Statistics 2019; Walsh and Kangaharan 2016). Despite well‐established evidence on the benefits of cardiac rehabilitation (CR) in reducing recurring cardiac events, improving quality of life and lowering mortality, access to and participation in CR remains alarmingly low among these groups (Thompson et al. 2009). A significant contributor to this disparity is the lack of culturally tailored programmes that address the unique needs, preferences and lived experiences of the diverse populations (Bulto and Hendriks 2025).

The current CR model in Australia is often delivered in clinical settings and designed around assumptions of cultural homogeneity. This one‐size‐fits‐all approach overlooks the diverse cultural, linguistic and structural barriers faced by priority populations. Migrants and refugees may contend with language difficulties, unfamiliarity with the healthcare system, stigma and cultural beliefs that influence health seeking behaviour (Bulto 2024). Indigenous Australians often face historical and ongoing mistrust in health care institutions and limited access to culturally safe care (Nolan‐Isles et al. 2021). Cardiac rehabilitation programmes that fail to incorporate cultural norms are not accessible.

Underutilisation of CR services by these populations perpetuates health disparities. For instance, Aboriginal and Torres Strait Islander peoples experience cardiac‐related hospitalisation at nearly twice the rate of non‐Indigenous Australians and are significantly less likely to attend CR (Australian Bureau of Statistics 2019; Brown 2012). Similarly, culturally and linguistically diverse (CALD) populations face a disproportionate burden of risk factors such as diabetes, hypertension and physical inactivity and have lower rate of CR enrolment and completion. Neglecting these disparities results in increasing premature death, healthcare utilisation and lost productivity.

Evidence indicates that culturally tailored CR interventions codesigned with communities and delivered in accessible, linguistically appropriate and culturally relevant ways can significantly improve engagement and outcomes (Joo and Liu 2021). Community‐based and home‐based CR models, the involvement of bicultural health professionals, and incorporation of traditional health practices and values have shown promise in small scale studies. However, such approaches remain underutilised, poorly funded and inconsistently implemented across Australia. There is a pressing need for health systems to invest in the development, evaluation and scaling up of culturally appropriate CR programmes, guided by meaningful community engagement.

Addressing this inequity requires systemic change. First, policy frameworks and funding mechanisms must prioritise equity in cardiac care by mandating the inclusion of culturally appropriate services in CR models. Second, health services must commit to co‐designing CR programmes with priority populations, ensuring cultural safety and relevance from the outset. Third, capacity‐building efforts are needed to train healthcare providers in cultural competence and to support the recruitment and retention of bicultural and Indigenous health workers. Finally, rigorous research and evaluation must underpin the development of these interventions, ensuring they are both effective and scalable.

Nurses play a central role in providing culturally tailored cardiac rehabilitation for culturally and linguistically diverse populations by ensuring care is responsive to patients' cultural, linguistic and social needs. This role encompasses conducting culturally sensitive assessments, adapting health education to align with patients' beliefs, values and health literacy levels, and facilitating effective communication through the appropriate use of professional interpreters and culturally appropriate resources. Nurses are also instrumental in fostering trust, promoting patient engagement and supporting shared decision‐making, which are critical for sustained participation in rehabilitation programmes. Additionally, nurses act as advocates and care coordinators, working collaboratively with multidisciplinary teams and community stakeholders to identify and address systemic and contextual barriers to access and continuity of care. Through these roles, nurses contribute to improving adherence, self‐management and rehabilitation outcomes, while advancing equity and reducing cardiovascular health disparities among underserved populations.

Healthcare leaders, researchers, policy makers and funding bodies in Australia have the responsibility and moral imperative to act. Ensuring equitable access to cardiac rehabilitation for all Australians is not only a matter of providing health service, but also a matter of ensuring equity. Without culturally tailored CR programmes, the health system will continue to leave behind those who need care the most.

## Funding

The author has nothing to report.

## Conflicts of Interest

The author declares no conflicts of interest.

## Data Availability

Data sharing not applicable to this article as no datasets were generated or analysed during the current study.

## References

[nop270536-bib-0001] Australian Bureau of Statistics . 2019. “Cardiovascular Disease, 2019.”

[nop270536-bib-0002] Brown, A. 2012. “Addressing Cardiovascular Inequalities Among Indigenous Australians.” Global Cardiology Science & Practice 2012, no. 1: 2. 10.5339/gcsp.2012.2. 25610833 PMC4239816

[nop270536-bib-0003] Bulto, L. 2024. “Voices Unheard: Bridging Language Gaps, Ensuring Equity and Inclusion of Non‐Native Speakers in Health Research and Clinical Trials.” Nursing Open 11, no. 9: e70048. 10.1002/nop2.70048. 39308320 PMC11417425

[nop270536-bib-0004] Bulto, L. , and J. M. Hendriks . 2025. “Cardiovascular Preventive Service Access Challenges Among African Immigrants: A Discussion Paper.” European Journal of Cardiovascular Nursing 24, no. 3: 483–487. 10.1093/eurjcn/zvae168. 39873688

[nop270536-bib-0005] Joo, J. Y. , and M. F. Liu . 2021. “Culturally Tailored Interventions for Ethnic Minorities: A Scoping Review.” Nursing Open 8, no. 5: 2078–2090. 10.1002/nop2.733. 34388862 PMC8363345

[nop270536-bib-0006] Nolan‐Isles, D. , R. Macniven , K. Hunter , et al. 2021. “Enablers and Barriers to Accessing Healthcare Services for Aboriginal People in New South Wales, Australia.” International Journal of Environmental Research and Public Health 18, no. 6: 3014. 10.3390/ijerph18063014. 33804104 PMC7999419

[nop270536-bib-0007] Thompson, S. C. , M. L. Digiacomo , J. S. Smith , et al. 2009. “Are the Processes Recommended by the NHMRC for Improving Cardiac Rehabilitation (CR) for Aboriginal and Torres Strait Islander People Being Implemented?: An Assessment of CR Services Across Western Australia.” Australia and New Zealand Health Policy 6: 29. 10.1186/1743-8462-6-29. 20042097 PMC2806388

[nop270536-bib-0008] Walsh, W. , and N. Kangaharan . 2016. “Aboriginal and Torres Strait Islander Cardiovascular Health 2016: Is the Gap Closing?” Heart, Lung & Circulation 25, no. 8: 765–767. 10.1016/j.hlc.2016.05.110. 27320852

